# The increasing rates of acute interstitial nephritis in Australia: a single centre case series

**DOI:** 10.1186/s12882-017-0747-7

**Published:** 2017-10-31

**Authors:** Gregory J. Wilson, Adrian L. Kark, Leo P. Francis, Wendy Hoy, Helen G. Healy, Andrew J. Mallett

**Affiliations:** 1 Department of Renal Medicine, Royal Brisbane and Women’s Hospital, Herston, QLD 4029 Australia; 2 CKD.QLD & NHMRC CKD.CRE, The University of Queensland, Herston, QLD Australia; 3 Department of Pathology, Royal Brisbane and Women’s Hospital, Herston, QLD Australia; 4 Centre for Chronic Disease, The University of Queensland, Herston, QLD Australia; 5 School of Medicine, The University of Queensland, Herston, QLD Australia

**Keywords:** Acute interstitial nephritis, Acute kidney injury, AIHW

## Abstract

**Background:**

The Australian Institute of Health and Welfare’s first report into acute kidney injury demonstrated a significant increase in the incidence of acute-tubulo interstitial nephritis, the ICD-10 code representing both acute interstitial nephritis and pyelonephritis, in women aged less than 55 years. In contrast, recent case series have reported rising rates of drug induced acute interstitial nephritis predominantly among elderly patients. Due to several limitations with the Australian Institute of Health and Welfare report, this new trend requires further investigation to determine if rates of acute interstitial nephritis are truly increasing among younger Australian women.

**Methods:**

Patients who underwent a renal biopsy at a single center from 2000 to 2015 were reviewed and those with biopsy confirmed acute interstitial nephritis were selected. Cause of acute interstitial nephritis, patient demographics, co-morbidities and renal indices for these patients when available were recorded and compared.

**Results:**

Eight hundred ninety-eight patients who underwent renal biopsy from 2000 to 2015 were reviewed and 40 patients were identified with biopsy confirmed acute interstitial nephritis. The rate of acute interstitial nephritis increased significantly over the study period (4 patients/2.2% of biopsies performed in 2000–03 vs. 19 patients/6.7% of all biopsies performed in 2012–15; *p* = 0.002). There was a marked increase in the number of women with AIN in the last four years of the study (2 patients and 2.1% of biopsies performed in women in 2000–2003 compared with 13 patients and 9.0% of biopsies performed in women in 2012–2015). Immune mediated causes of acute interstitial nephritis and NSAID associated AIN were more common in women (9 females vs. 3 males), occurred more frequently in the last eight years of the study and predominantly in patients under 55 years of age.

**Conclusions:**

Our study demonstrates a significant increase in the number of patients with biopsy confirmed AIN. Also, we provide preliminary evidence in support of an increase in rates of younger women with immune mediated acute interstitial nephritis. These results support the findings of the Australian Institute of Health and Welfare and suggest that younger women may be at higher risk of immune mediated and NSAID associated acute interstitial nephritis.

## Background

Acute interstitial nephritis (AIN) is an immune mediated condition that is characterized by an inflammatory infiltrate in the kidney interstitium and is a well-recognized cause of acute kidney injury (AKI). The incidence of AIN is increasing worldwide and previous studies have ascribed this to a surge in drug induced AIN in elderly patients [1–5]. The Australian Institute of Health and Welfare (AIHW) recently released their first national report into acute kidney injury [6]. That snapshot reviewed hospital ICD-10 coding to assess the causes and incidence of AKI over the last 15 years in Australia. Surprisingly, this showed that since 2008 there has been a significant increase in the incidence of acute tubulo-interstitial nephritis (the ICD-10 code for both AIN and acute pyelonephritis) in women less than 55 years of age with acute tubulo-interstitial nephritis now representing the most common cause of hospitalization from AKI in women in this age group [6]. An increased risk of AIN in younger women has not been previously reported and the Australian experience is an emerging phenomenon at odds with previous findings of increasing rates of AIN in elderly patients.

Before these conclusions can be made, however, several limitations in the AIHW report must be addressed by further research. Specifically, ICD-10 coding is imprecise, with AIN and pyelonephritis included as one ICD-10 code. As such the AIHW report is unable to determine which of these two entities is increasing. A case series of patients with biopsy-confirmed AIN would provide preliminary evidence to investigate if the new trends reported by the AIHW are being driven by increasing rates of AIN in younger women. Furthermore, while the AIHW report recorded patient age and gender it was not able to record other key patient factors, such as the causes of acute tubulo-interstitial nephritis, which might underlie their reported trend.

The present study seeks to address the limitations of the AIWH report by examining a cohort of patients with biopsy confirmed AIN in a single center to explore (a) whether there has been an increase in the rates of AIN and when this change began, (b) whether there was a disproportionate rise in AIN in younger women and (c) the causes, characteristics and outcomes of patients with AIN.

## Methods

Design and implementation of this single-center retrospective observational cohort study was subject to institutional human research ethics committee review (HREC/15/QRBW/544) as a quality assurance and clinical audit activity. The study was undertaken at the Royal Brisbane and Women’s Hospital, a tertiary referral hospital that undertakes all public renal biopsies for northern Brisbane and surrounding regional hospitals in Queensland, with a catchment area of approximately one and a half million people.

**Fig. 1:**
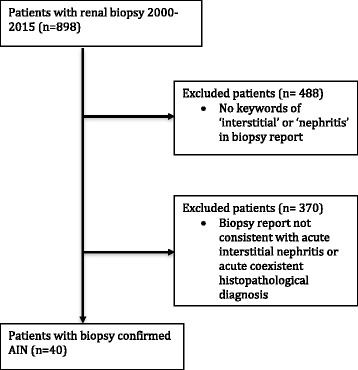
Flow diagram of patient selection process

Figure 1 describes the patient selection process. Adults (18 years and over) with AIN were identified through a database search of all patients from the Royal Brisbane and Women’s Hospital who underwent renal biopsy from the 1st of January 2000 until the 31st of December 2015 using the keywords ‘Interstitial’ and ‘Nephritis’ within kidney biopsy reports. Biopsy results were manually reviewed to identify patients with a histological diagnosis of AIN. AIN was defined as the presence of an inflammatory infiltrate within the interstitium with or without tubulitis. The diagnosis was determined by two expert kidney histopathologists who were continuously employed over the study period. The degree of interstitial infiltration, type of cellular infiltrate, the presence of tubulitis and immunofluorescence were recorded. Patients who had a second coexistent histological diagnosis at the time of biopsy of either acute tubular necrosis or acute glomerulonephritis were excluded. Patients with positive urine cultures were also excluded. This was to ensure that only patients with a single diagnosis of AIN and no other acute pathology were included.

Medical records of the patients were reviewed. Patient demographics (age, gender, date of death, co-morbidities), AIN characteristics (presumed cause as determined by the treating nephrologist, treatment, renal replacement therapy, co-existent disease on biopsy) and renal indices (serum creatinine at biopsy, urine white cells, urinary eosinophils, urine protein creatinine ratio, Acute Kidney Injury Network stage) were recorded. When available, serum creatinine levels two weeks post biopsy and final serum creatinine measured were recorded if they were at least three months after the initial biopsy. When available, serum creatinine levels three to twelve months prior to biopsy were also recorded for a baseline serum creatinine.

The AIN etiology was categorized into 6 groups based on a classification system used in previous case series [1]: antibiotic, non-steroidal anti-inflammatory drugs (NSAIDs), proton pump inhibitors (PPIs)/ H2 antagonists, immune mediated diseases (including tubulointerstitial nephritis and uveitis), other causes and unknown cause. Patients were assigned to the unknown cause group when the cause of AIN had not able to be determined by the treating team. Treatment administered was categorized into steroid therapy or no steroid therapy. No distinction was made between different formulations of steroids or routes of administration. Urine white cells were classified into three categories of less than 10 WCC per high-powered field (HPF), 10–100 WCC/HPF and more than 100 WCC/HPF. The Acute Kidney Injury Network (AKIN) stage was calculated using the serum creatinine at the time of biopsy divided by the serum creatinine measured in the 3–12 months prior to biopsy.

Data processing and statistical analyses were performed using R (3.2.4, R Foundation for Statistical Computing, Vienna, Austria) [7]. Dichotomous variables were displayed as counts with percentages; categorical variables were displayed as counts with relative frequencies (%). Continuous variables were assumed to be non-parametric and therefore were displayed as medians with interquartile ranges. Pearson Chi Square test of best fit was used to determine differences in categorical variables. Differences in serum creatinine at different time points between patient subgroups were assessed using Mann-Whitney U test for dichotomous variables and Kruskal-Wallis test for categorical variables.

## Results

Patient demographics and co-morbidities are outlined in Table 1. Biopsy confirmed acute interstitial nephritis was identified in only 40 patients (4.4% of all biopsy performed), of whom 55% were female. Although this relatively small sample size limits both the statistical power of this study and the conclusions that can be drawn from the analyses, the data will nevertheless be explored in order to characterize emerging trends in biopsy confirmed AIN within a single Australian center over a 16 year period.

**Table 1:** Patient Characteristics and Comorbidities

	Male	Female	Total
N	18 (45%)	22 (55%)	40
Age at biopsy (IQR)	59.7 yrs. (42.5–78.0)	58.2 yrs. (37.8–67.0)	58.6 yrs. (40.3–74.5)
Comorbidities	(%)	(%)	(%)
Ischaemic Heart Disease	5 (28)	1 (5)	6 (15)
Hypertension	8 (44)	8 (36)	16 (40)
Dyslipidaemia	4 (22)	5 (23)	9 (22.5)
Diabetes Mellitus (type 1 or 2)	7 (39)	5 (23)	12 (30)
Autoimmune disease	4 (22)	6 (27)	10 (25)
PPI use	6 (33)	7 (32)	13 (32.5)
Cancer (active or in remission)	7 (39)	4 (18)	11 (27.5)
Mental Health diagnosis	2 (11)	6 (27)	8 (20)

Patient co-morbidities were similar across both genders. Patients with immune-mediated diseases including tubulo-interstitial nephritis with uveitis (TINU) as the cause of their AIN were younger than those with other causes of AIN (median age 40.7 years vs. 63.9 years in patients with other causes of AIN) however, this did not reach significance. Patients with proton pump inhibitor or H2 antagonist (PPI/H2) use and antibiotic use as the cause of their AIN were older than those with other causes (median age 72.3 years and 66.8 years respectively). Neither gender nor co-morbidities were significantly different by age.

**Fig. 2:**
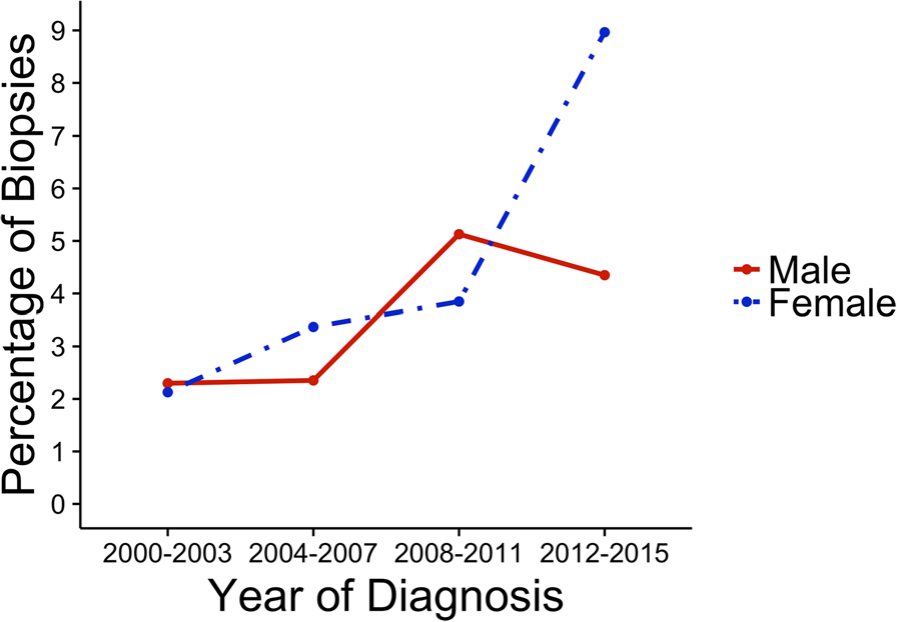
Percentage of Renal Biopsies with AIN by Year of Diagnosis

The numbers of patients with AIN were compared over 4 year intervals. There was an increase in the number of patients with biopsy proven AIN over the study period with only four patients identified in 2000 to 2003 compared with nineteen patients from 2012 to 2015 (*p* = 0.002). There was also an increase in the percentage of patients with AIN as a proportion of all patients who underwent renal biopsy, with 2.2% of all patients with a renal biopsy diagnosed with AIN in 2000 to 2003 compared to 6.7% of patients with a renal biopsy in 2012–2015. There was an increase in the number of both male and female patients with AIN during the study. Fig. 2 describes the increase in AIN in men and women as a percentage of the total number of men and total number of women who underwent renal biopsies over the study period. In the last four years of the study there was marked increase in the percentage of women with AIN on renal biopsy (2 patients and 2.1% of all biopsies performed in women in 2000–2003 compared with 13 patients and 9.0% of all biopsies performed in women in 2012–2015) and a less marked increase in men with AIN (2 patients and 2.3% of all biopsies performed in men in 2000–2003 compared with 6 patients and 4.3% of all biopsies performed in men in 2012–2015).

### AIN characteristics and patient outcomes

Table 2 describes the renal indices at time of biopsy. As expected, serum creatinine values were raised in the majority of patients (95%) at the time of diagnosis. Serum creatinine values were higher in males compared to females though this was not significant. Patients with antibiotics as the cause of their AIN had higher elevations in serum creatinine while those with NSAID and immune mediated causes of AIN had lower serum creatinines. The majority of patients had sterile pyuria and proteinuria with varying degrees of severity. Urinary eosinophils were only tested in a minority of patients.

**Table 2:** Renal Indices at time of Biopsy

Renal Indice	
Urine White Cells (per HPF)	(%)
< 10	11 (27.5)
10–100	24 (60.0)
100<	5 (12.5)
Urinary Eosinophils	(%)
Present	3 (7.5)
Absent	4 (10)
Not tested	33 (82.5)
Urine Protein Creatinine Ratio	(%)
Less than15 mg/mmol Cr	5 (12.5)
More than15 mg/mmol Cr	22 (55)
Not tested	13 (32.5)
Co-existent Disease on Biopsy	
Hypertensive Nephrosclerosis	6 (15)
Diabetic Nephropathy	4 (10)
Serum Creatinine (μmol/L)	(IQR)
All patients	246 (164–350)
Gender	
Male	261 (207–419)
Female	228 (138–333)
P	0.24
Presumed Cause of AIN	
Unknown (*n* = 14)	240 (164–439)
Antibiotic (*n* = 7)	430 (274–454)
Non-Steroidal Anti-Inflammatory Drugs	219 (117-248)
(NSAIDS) (*n*=5)	
PPI/H2 antagonist (*n*=4)	289 (245-332)
Immune mediated diseases (*n*=7)	178 (125-217)
Other (*n*=3)	388 (248-473)
P	0.16
Treatment	
Steroid therapy	237 (164-332)
No treatment given	260 (164-430)
P	0.59
Co-existent Disease on Biopsy	
Hypertensive Nephrosclerosis	330 (171-436)
Diabetic Nephropathy	244 (168-245)
No co-existent disease	242 (162-347)
P	0.55
AKIN Severity (*n*=12)*	
1 (7)	164 (140-210)
2 (3)	197 (155-268)
3 (6)	380 (286-442)
P	0.01

*Only 12 patients had serum creatinine measured 3-12 months prior to renal biopsy

All patients had evidence of an interstitial infiltrate on renal biopsy histology. Lymphocytes were present in the interstitial infiltrate in all patients while eosinophils were seen in 32 patients, plasma cells in 17 patients and neutrophils were noted in only 3 patients. Tubulitis was present in 38 of the 40 patients. Immunofluorescence was negative in the majority of patients (30/40). Five patients had 1–2+ IgG staining which was linear in the glomerular and tubular basement membrane in four patients and diffusely distributed in the remaining patient. Those patients with linear IgG staining had other histological findings consistent with diabetic nephropathy and the patient with diffuse IgG staining did not have a cause of AIN identified. 4 patients had trace-1+ IgM staining within the mesangium and 3 of these patients had a drug induced cause of AIN (antibiotics or hydrochlorothiazide use) while the cause of AIN in the other patient was not identified. One patient had 2+ IgA staining in the mesangium and the cause of AIN in this patient was NSAID use.

Table 3 describes the presumed causes of AIN. Most commonly, no cause was identified as the precipitant for AIN (unknown cause). Antibiotic use was the second most frequently reported cause of AIN (7, 17.5%), with penicillin as the most common antibiotic class to precipitate AIN (4, 10%). Tubulo-interstitial nephritis with uveitis (4, 10%) was the most common immune mediated cause followed by sarcoidosis (2, 5%) and Sjogren’s Syndrome (1, 2.5%). Other causes included a patient with oxalate nephropathy, a patient with hydrochlorothiazide use and a patient with lamotrogine use as the precipitant. Compared with men, women were more likely to have NSAID associated AIN (4 females vs. 1 male) and immune mediated AIN (5 female vs. 2 male). Immune mediated AIN and NSAID associated AIN occurred only in patients from 2008 onwards.

**Table 3:** AIN Etiology

Presumed Cause of AIN	Total (%)	Male (%)	Female (%)
Unknown	14 (35)	10 (56)	4 (18)
Antibiotic	7 (17.5)	3 (17)	4 (18)
Penicillin	4 (10)	1 (6)	3 (14)
Ciprofloxacin	1 (2.5)	1(6)	0 (0)
Nitrofurantoin	1 (2.5)	0 (0)	1 (5)
Vancomycin	1 (2.5)	1 (6)	0 (0)
NSAID	5 (12.5)	1 (6)	4 (18)
PPI/H2 antagonist	4 (10)	1 (6)	3 (14)
Immune mediated diseases	7(17.5)	2 (11)	5 (23)
TINU	4 (10)	1 (6)	3 (14)
Sarcoidosis	2 (5)	1 (6)	1 (5)
Sjogren’s Syndrome	1 (2.5)	0 (0)	1 (5)
Other	3 (7.5)	1 (6)	2 (9)

Over half the patients received steroid treatment (23 patients; 57.5%). The majority of these were given a course of high dose oral prednisone (19 patients; 83%), while three were given intravenous methylprednisone induction followed by high dose oral prednisone and one was given high dose prednisone and azathioprine. Men and women were treated at similar rates with 12 women (55%) and 11 men (61%) receiving treatment. The majority of patients with either NSAID (4/5) or immune mediated AIN (6/7) received treatment while those with antibiotic use, PPI/H2 use or an unknown cause of AIN were less likely to receive steroid therapy (7/14, 3/7 and 2/4 patients, respectively). Patients who received steroid treatment had on average lower serum creatinines compared to those who did not receive treatment. The majority of patients who did not receive treatment had evidence of interstitial fibrosis and tubular atrophy on biopsy (13 patients; 76%) compared to patients who did receive therapy (5 patients 21%) and this may explain the difference in the median serum creatinine between the two groups.

Sixteen patients had serum creatinines measured in the 3–12 months prior to biopsy. AKIN stage was calculated for these patients and as expected the serum creatinines by stage were significantly different.

Twenty-eight patients had a measured serum creatinine two weeks post biopsy (see Table 4). The degree of creatinine elevation did not differ by gender, cause of AIN, treatment given, co-existent disease on biopsy or urinary indices. At two weeks post biopsy the majority of patients had an improvement in serum creatinine (79%), and this was not modulated by patient gender, cause of AIN or co-existent disease on biopsy. The AKIN stage of AKI did not affect the rate of renal recovery, with minimal difference in serum creatinine at two weeks post biopsy between stages.

**Table 4:** Median serum creatinine (μmol/L), delta serum creatinine improvement and the percentage of patients with decreased serum creatinine at two week post biopsy

*N* = 28	Median SCr at 2 weeks (IQR)	Decrease in SCr at 2 weeks (IQR)	Percentage patient with decreased SCr at 2 weeks (%)
All patients (28)	136 (112–197)	93 (14–158)	79
Gender			
Male (14)	133 (112–185)	123 (87–170)	86
Female (14)	141 (119–119)	58 (4–104)	71
P	0.66	0.18	
Comorbidities			
Ischaemic Heart Disease (5)	193 (159–209)	124 (68–152)	100
Hypertension (10)	129 (113–163)	110 (6–209)	70
Dyslipidaemia (7)	193 (133–265)	88 (47–138)	86
Diabetes Mellitus (type 1 or 2) (9)	170 (159–290)	88 (30–124)	78
Autoimmune conditions (6)	129 (113–141)	44 (113–141)	67
PPI use (10)	173 (115–244)	91 (51–170)	90
Cancer (9)	129 (112–193)	90 (3–176)	78
Mental and Behavioural condition (5)	208 (142–290)	96 (68–188)	100
Presumed Cause of AIN			
Unknown (9)	170 (114–248)	90 (−3–146)	67
Antibiotic (5)	127 (112–131)	176 (68–303)	100
Non-Steroidal Anti-Inflammatory Drugs (NSAIDS) (3)	139 (128–149)	88 (43–105)	67
PPI/H2 antagonist (4)	184 (129–245)	72 (39–130)	100
Immune mediated diseases (6)	119 (99–136)	90 (16–104)	67
Other (1)	314	241	100
P	0.29	0.43	
Treatment			
Steroid therapy (16)	135 (110–159)	189 (14–158)	81
No treatment given (12)	154 (113–218)	102 (34–170)	75
P	0.59	0.58	
Co-existent Disease on Biopsy			
Hypertensive Nephrosclerosis (3)	157 (143–176)	124 (64–214)	100
Diabetic Nephropathy (3)	170 (116–231)	–3 (−4–21)	33%
No co-existent disease (22)	134 (112–196)	96 (36–170)	82
P	0.79	0.15	
AKIN Severity (*n* = 13)			
1 (5)	159 (94–170)	3 (−6–18)	60
2 (3)	119 (115–204)	119 (115–204)	67
3 (5)	112 (102–127)	228 (176–303)	100
P	0.65	0.01	
Renal Replacment Therapy		N (%)	
RRT at two weeks		2 (5)	
RRT at 3 months or later		3 (7.5)	

Twenty-five patients had a final serum creatinine measured at least 3 months after biopsy. There was significant variation between the time of biopsy and final recorded serum creatinine that limited analysis (range 0.3–9.6 years from date of biopsy). The median serum creatinine on the last recorded measure was 99 μmol/L (IQR 90–135). The median serum creatinine on the last recorded serum creatinine was lower in patients who had received steroid therapy (92 μmol/L; IQR 76–107) compared to those who did not receive therapy (116 μmol/L; IQR 101–127) though this difference did not reach significance. Two patients required renal replacement therapy (RRT) at two weeks and three patients (including those at two weeks) required long term RRT. Two of the three patients who required long term RRT had co-existent CKD on renal biopsy.

Eight patients died over the course of the 16-year period reviewed. The cause of death was not recorded in the clinical record. The median age at death was 66.2 years (IQR 55.2–75.4). Two patients died within 2 weeks of their diagnosis. The median time to death from biopsy was 3.0 years (IQR 0.4–7.2). Two of the deceased patients had required long term RRT prior to their death. There was an equal distribution of gender in these patients. The most common identified cause of AIN in patients who died was antibiotics associated AIN (3 patients) however 5 patients with an unknown cause of AIN also died. These patients had significantly higher rates of hypertension (87.5% deceased vs. 39% alive; *p* < 0.001). Rates of other co-morbidities were similar.

## Discussion

The present study was the first to investigate rates of biopsy confirmed AIN in Australia and, consistent with the findings reported by the AIHW [6], it identified a significant increase in the number of patients diagnosed with acute interstitial nephritis over the 16-year period reviewed. Importantly, our findings are also suggestive of a recently emerging increase in the number of young females with biopsy proven AIN; from 2008 onwards all patients with immune mediated AIN were under the age of 55 (median age 40.7 yrs) and the majority were women (71%). This is in line with the findings described by the AIHW, who noted an increase in acute tubulo-interstitial nephritis in women less than fifty-five years of age. Indeed, the time period over which rates of immune mediated AIN increased (0 patients from 2000 to 2007 vs 7 patients from 2008 to 2015) is similar to that described in the AIHW report. Due to the small sample size of the current study, however, further research exploring larger cohorts over longer time-periods is required to corroborate this trend.

There are several possible explanations for the rising incidence of immune mediated AIN in younger women seen in our study. Firstly, multiple studies have shown that rates of autoimmune diseases worldwide (including causes of AIN such as Sjogren’s syndrome and sarcoidosis) are increasing [8]. This trend is seen nationally in Australia with increasing incidence of asthma, food allergies and anaphylaxis over the last 20 years [9]. Secondly, autoimmune conditions are more common in women compared to men [10] and an increasing incidence of immune mediated AIN would have a more noticeable effect on the number of women diagnosed with AIN. Thirdly, immune conditions associated with immune mediated AIN such as TINU, sarcoidosis or Sjogren’s syndrome are more common in younger women and an increase in these conditions would likely lead to a disproportionate increase in women developing AIN.

The current findings also confirm that while AIN can occur in any age group, increased use of NSAIDS, antibiotics and PPIs combined with higher rates of renal impairment put elderly patients at greater risk [11]. Specifically, patients with PPI/H2-induced AIN and antibiotic-induced AIN tended to be older (median age 72.3 years and 66.8 years, respectively). Patients with NSAID use were predominantly female (80%) and younger than other patients with a drug induced AIN with a median age of 60.3 yrs. Also, NSAID associated AIN only occurred from 2008 onwards and this trend may also be contributing to the increase in AIN in younger women seen in our study and by the AIHW. It has been decades since NSAID use among young Australian women was linked with kidney injury [12], with more recent Australian studies showing that NSAID use in Australia is more common in elderly patients [13, 14]. In contrast, our findings suggest a reversion to previous community behaviors of analgesic overuse among younger, predominantly female cohorts.

The majority of AIN patients have a benign course and a complete or partial renal recovery [1, 11]. Although follow-up data was only available for some of the patients in this study, it indicated that most patients had a marked improvement in serum creatinine at two weeks (median sCr 136 μmol/L) and near complete renal recovery on the final serum creatinine measured (median sCr 99 μmol/L). This improvement was independent of age, gender, patient co-morbidity or cause. Only 3 patients required RRT, and two of these had pre-existing CKD.

The treatment of AIN is largely empiric, with withdrawal or treatment of the causative agent the most effective intervention [1]. Traditionally, a course of high dose oral prednisone (with or without intravenous methylprednisone) has been prescribed, particularly in patients with an idiopathic or immune mediated cause. Reports on the effectiveness of steroids in both these and drug induced AIN is unproven and based on evidence from uncontrolled, retrospective studies [1, 2, 5, 11]. The present findings corroborate these previous results; steroid treatment did not make a significant difference in renal function at two weeks and on the final serum creatinine measured. However, follow-up data was available for a proportion of our patient cohort and conclusions should be tentatively drawn.

In addition to the sample size issues already discussed, other factors should be taken into consideration when interpreting the present results. Being a single center study it is possible that our findings are not representative of national trends. Further investigations across multiples centers nationally are necessary in order to obtain a representative Australian sample. Patients with mild AKI or a typical presentation of AIN may not have undergone renal biopsy and thus may be underrepresented in our findings. By only reviewing patients who have biopsy proven AIN we include only a small fraction of those who are clinically diagnosed with AIN, as those with less severe AKI do not generally proceed to renal biopsy. The complement of nephrologists changed over the course of the study and the decision to undertake a renal biopsy was not standardized. Referring patterns from other specialists and clinics may have changed over the course of the study and could have contributed to an increase in the incidence of AIN. And lastly, our study excluded patients with pyelonephritis. Because of this, we were unable to compare rates of AIN to those of acute pyelonephritis and thus our findings cannot rule out the possibility that the increase in acute tubulo-interstitial nephritis reported by the AIHW was due to increased rates of both acute pyelonephritis *and* AIN. Further investigations are required to adjudicate this possibility.

## Conclusions

Our study demonstrates a significant increase in AIN over the last 16 years and supports the AIHW’s finding of increasing rates of AIN in Australia. While rates of both men *and* women with AIN were shown to increase in our study, a trend of increasing rates of younger women with immune mediated AIN and NSAID associated AIN, notably in the study’s last eight years, was also observed. Although preliminary, these results support the trends identified by the AIHW and suggest that younger women may have a heightened risk of both immune mediated and NSAID induced acute interstitial nephritis.

## Abbreviations

AIHW: Australian Institute of Health and Welfare
AIN: Acute interstitial nephritis
AKI: Acute kidney injury
AKIN: Acute kidney injury network
NSAID: Non steroidal anti-inflammatory drugs
PPI: Proton pump inhibitors
RRT: Renal replacement therapy
TINU: Tubulo-interstitial nephritis and uveitis
